# *ModelCraft*: an advanced automated model-building pipeline using *Buccaneer*

**DOI:** 10.1107/S2059798322007732

**Published:** 2022-08-25

**Authors:** Paul S. Bond, Kevin D. Cowtan

**Affiliations:** a Department of Chemistry, University of York, York YO10 5DD, United Kingdom; MRC Laboratory of Molecular Biology, United Kingdom

**Keywords:** *ModelCraft*, X-ray crystallography, structure solution, model building, automation, software, *Buccaneer*

## Abstract

Interactive model building can be a difficult and time-consuming step in the structure-solution process. Automated model-building programs such as *Buccaneer* often make it quicker and easier by completing most of the model in advance. However, they may fail to do so with low-resolution data or a poor initial model or map. The *Buccaneer* pipeline is a relatively simple program that iterates *Buccaneer* with *REFMAC* to refine the model and update the map. A new pipeline called *ModelCraft* has been developed that expands on this to include shift-field refinement, machine-learned pruning of incorrect residues, classical density modification, addition of water and dummy atoms, building of nucleic acids and final rebuilding of side chains. Testing was performed on 1180 structures solved by experimental phasing, 1338 structures solved by molecular replacement using homologues and 2030 structures solved by molecular replacement using predicted *AlphaFold* models. Compared with the previous *Buccaneer* pipeline, *ModelCraft* increased the mean completeness of the protein models in the experimental phasing cases from 91% to 95%, the molecular-replacement cases from 50% to 78% and the *AlphaFold* cases from 82% to 91%.

## Abstract 2

*ModelCraft* is a new model-building pipeline which improves on previous *Buccaneer* pipelines by the addition of new steps that make it more likely to build a complete protein model, especially when starting from a poor molecular-replacement solution.

## 1. Introduction

*Buccaneer* is a program for automated protein model building that was developed to be effective even when the data resolution is limited (Cowtan, 2006 ▸). Main-chain tracing works by identifying C^α^ positions with an oriented electron-density likelihood function that was originally developed under the name *FFFear* (Cowtan, 2001 ▸). The likelihood targets are generated using a map for a known reference structure that has been modified to have the same resolution, scale and noise level as the map to be searched. The method is fast and is able to build at a range of resolutions, but may fail when the starting phases are inaccurate. The main-chain tracing algorithm was subsequently expanded into a full model-building program with sequencing, side-chain building and expansion of noncrystallographic symmetry (NCS) copies (Cowtan, 2008 ▸).

*Buccaneer* does not perform any global refinement of coordinates or *B* factors, so it works best when combined with a refinement program such as *REFMAC* (Kovalevskiy *et al.*, 2018 ▸) in a model-building pipeline. This improves the model geometry and fit to density, and produces an updated map that can be used in the next building cycle. *Buccaneer* and model-building pipelines that include *Buccaneer* are distributed with the *CCP*4 software suite (Winn *et al.*, 2011 ▸). The oldest *Buccaneer* pipeline is that available via the *CCP*4*i* graphical user interface (GUI; Potterton *et al.*, 2003 ▸). This pipeline, which is also available via *CCP*4 Cloud (Krissinel *et al.*, 2018 ▸), runs five cycles of *Buccaneer* followed by *REFMAC* and outputs the model from the final cycle.

Until recent versions, the *Buccaneer* pipeline in the newer *CCP*4*i*2 GUI (Potterton *et al.*, 2018 ▸) was largely a reimplementation of the *CCP*4*i* pipeline. It now runs up to 25 cycles by default and will stop automatically if the model is not improving. It also carries out preliminary shift-field refinement of molecular-replacement models using *Sheetbend* (Cowtan *et al.*, 2020 ▸), adds water molecules using *Coot* (Emsley *et al.*, 2010 ▸) once *R*_work_ is better than 40% and outputs the model from the cycle with the lowest *R*_free_. The *CCP*4*i*2 pipeline additionally provides the option of including pruning steps, which use two neural networks trained to predict main-chain and side-chain correctness (Bond *et al.*, 2020 ▸).

*CCP*4*Build* is a newer model-building pipeline available in *CCP*4 Cloud that combines *Parrot* (Cowtan, 2010 ▸), *Buccaneer*, *REFMAC*, *Coot* and *EDSTATS* (Tickle, 2012 ▸). At the start of each cycle, it runs *Buccaneer* and *REFMAC* with and without *Parrot* density modification beforehand and the result with the best *R* factors is chosen. It also has steps for trimming the model using the real-space *Z*_diff_ (RSZD) metric from *EDSTATS*, reconstructing the side chains, fitting the protein and performing real-space refinement in *Coot* and adding waters if the model and data resolution are good enough. These steps can be turned on or off, or they can be attempted with the result only being accepted if there is a reduction in the *R* factors. The pipeline terminates if *R*_free_, the number of residues built or the electron-density correlation coefficient (EDCC) have not improved in a set number of cycles.

*Buccaneer* is also used in the final stages of *CRANK*2 (Skubák & Pannu, 2013 ▸), which is a larger structure-solution pipeline for experimental phasing with SAD, MAD, SIRAS and MR-SAD. Refinement is carried out using a multivariate probability function that simultaneously combines experimental phase information with information from density modification and model building. More recently, this approach was extended to include rebuilding from an initial partial model (Skubák *et al.*, 2018 ▸), which can aid completion with low-resolution data or a weak anomalous signal.

*CAB* (Burla *et al.*, 2018 ▸) is a model-building pipeline in the *SIR* suite (Burla *et al.*, 2015 ▸) that uses *Buccaneer*. It starts with a phase-refinement procedure that combines classical density modification using *DM* (Cowtan *et al.*, 2001 ▸) with other phase-refinement techniques (Burla *et al.*, 2017 ▸). This is followed by repeated runs of the *CCP*4*i* *Buccaneer* pipeline with a weighted combination of the input and output phases. The cycle terminates once there is an increase in *R*_free_, subject to *R*_free_ and sequence coverage reaching acceptable thresholds. The model with the lowest *R*_free_ is chosen for final refinement and heavy atoms, if present, are added into the largest peaks. If the output model does not have sufficient sequence coverage then the pipeline is repeated with modified input phases. A newer *CAB* II pipeline has been released that builds nucleic acid structures using *Nautilus* (Cowtan, 2014 ▸) with an updated library of backbone conformations (Cascarano & Giacovazzo, 2021 ▸).

There are a number of other model-building programs with alternative approaches to *Buccaneer*. *ARP*/*wARP* works by adding, refining and interpreting dummy atoms (Langer *et al.*, 2008 ▸). Although this procedure by itself performs better with high-resolution data, a newer method of superposing fragments from homologous structures greatly improves model completion at low resolution (Chojnowski *et al.*, 2020 ▸). *Phenix AutoBuild* (Terwilliger *et al.*, 2008 ▸) locates α-helices and β-strands and extends them using a library of tripeptide fragments. The pipeline builds and merges multiple models and iterates model building with statistical density modification using *RESOLVE* (Terwilliger, 2000 ▸). *SHELXE* (Sheldrick, 2010 ▸) also uses α-helix, β-sheet and tripeptide templates. It has a cautious approach to backbone tracing that make it useful for improving poor phases or extending a partial molecular-replacement model.

This paper presents a new model-building pipeline called *ModelCraft*. It incorporates the as-yet unpublished additions to the *CCP*4*i*2 *Buccaneer* pipeline with newer developments such as density-modification methods and the use of *Nautilus* for building nucleic acids as well as protein. It is written as a Python module and command-line script so that the same pipeline can be shared between multiple user interfaces.

## 2. ModelCraft

### 2.1. Overview

The overall *ModelCraft* pipeline proceeds as follows. If a starting model is provided, it is refined with both shift-field and conventional refinement. A single cycle of *ModelCraft* then consists of the following seven steps.

(i) Protein chain, residue and side-chain pruning.
(ii) Density modification.
(iii) Dummy-atom addition.
(iv) Protein building.
(v) Protein-chain pruning.
(vi) RNA and DNA building.
(vii) Water addition.

By default the pipeline runs for a maximum of 25 cycles, but it stops automatically if *R*_free_ at the end of the cycle has not improved over its previous best value for four cycles. The model from the cycle with the lowest *R*_free_ is chosen as the output. If the resolution is better than 2.5 Å and *R*_work_ for the output model is better than 30%, then side chains that are missing or predicted to be incorrect are rebuilt.

### 2.2. Shift-field refinement

A starting model can be provided as input to the pipeline, which could be from molecular replacement of a homologue or a predicted model. As regions of the model may need large concerted shifts in order to fit the density, *Sheetbend* is used for preliminary shift-field refinement, which has a larger range of convergence than conventional refinement (Cowtan *et al.*, 2020 ▸). 12 cycles of *Sheetbend* are used with the resolution increasing linearly from 6 to 3 Å, followed by ten cycles of *REFMAC*. All *REFMAC* refinement in *ModelCraft* is carried out with automatic restraint weighting and no H atoms, as this was found to give improved performance in previous *Buccaneer* pipelines.

### 2.3. Pruning

The protein model is pruned using two neural networks trained to predict main-chain and side-chain correctness for individual residues (Bond *et al.*, 2020 ▸). Protein chains of up to 20 residues are removed if the mean main-chain correctness for the chain is less than 0.2 times the median main-chain correctness for the whole structure. This occurs at the start of the cycle and after building the protein using *Buccaneer*. The pruning step at the start of the cycle additionally removes individual residues and side chains if the main-chain or side-chain correctness is less than 0.5 times the median. However, this is not performed in the first cycle or if the data do not extend beyond 2.3 Å resolution. A maximum of 20% of the residues or side chains are pruned at each stage. Both pruning steps are followed by five cycles of refinement with *REFMAC*.

### 2.4. Density modification

The second step in a cycle of *ModelCraft* is to modify the density through solvent flattening, histogram matching and noncrystallographic symmetry (NCS) averaging using five cycles of *Parrot* (Cowtan, 2010 ▸). If the number of copies of the molecule in the asymmetric unit is not known then it is estimated from the Matthews probability (Kantardjieff & Rupp, 2003 ▸) in order to calculate the solvent content. The NCS operators are determined using the current model and the density is averaged using multiple pairwise weighted masks. However, the model is not used for calculation of the solvent mask as this does not work well with partial or fragmented models. Instead, both the solvent mask and NCS masks are recalculated during each *Parrot* cycle using the current map.

### 2.5. Dummy-atom addition

The next step is to add dummy atoms into the *Parrot* map using the *Coot* *findwaters* program with the ‘flood’ option. Dummy O atoms are added into peaks with heights above two standard deviations (σ) that are within a 1.9–10 Å distance of the model, with the distance between dummy atoms being at least 1.4 Å. The hybrid model with dummy atoms added is then refined using ten cycles of *REFMAC*, but the result is only accepted if it has a better *R*_free_ factor than the previous refinement of the model without dummy atoms. If there is no improvement in *R*_free_ then the map from *Parrot* will be used in subsequent building steps. It is important to use *R*_free_ to assess the improvement, as *R*_work_ will almost certainly decrease owing to overfitting with the increased number of parameters being refined and the lack of restraints on the dummy atoms. Dummy atoms and water atoms are discarded after this refinement as they are only used for phase improvement and not for map interpretation.

### 2.6. Model building

Protein building is performed using *Buccaneer*. Similar to previous *Buccaneer* pipelines, *ModelCraft* uses three cycles of *Buccaneer* in the first iteration and two cycles in subsequent cycles. The map for building into is calculated after anisotropy correction, without the free reflections and up to a 2 Å resolution limit. If there is a current model it is passed to *Buccaneer*, which will keep nonprotein residues fixed and not build within 1 Å of them. Two *Buccaneer* options, referred to as fast mode and correlation mode, are used. Fast mode alters the finding step to search for three-residue α-helices and β-strands instead of using a full *FFFear* search. Correlation mode makes the log-likelihood (LLK) target invariant to the scale and offset of the map. Additionally, the starting model passed to *ModelCraft* is used as a source of additional residue positions in the finding step. Every third residue is taken from the model after removing residues with LLK scores lower than two standard deviations below the mean.

Nucleic acids are built using three cycles of *Nautilus*, which is a companion program to *Buccaneer* for nucleic acid building. It is important to prune protein chains before this because *Buccaneer* is likely to build into the RNA/DNA density and *Nautilus* will keep residues other than nucleic acids fixed and avoid building into them. The protein and nucleic acid building steps are both followed by ten cycles of *REFMAC*.

### 2.7. Water addition

Waters are added using the *Coot findwaters* program without the ‘flood’ option. This builds waters into peaks with heights above 2σ and volumes less than 15 Å^3^, provided that the peak is further than 2.4 Å from other atoms and within 3.2 Å of an N or O atom. This is repeated three times so that outer-shell water molecules with hydrogen bonds to waters added earlier can be included. The model with waters is refined using ten cycles of *REFMAC*. As with dummy atoms, the refined model is only accepted if it has a better *R*_free_ than the previous refinement without waters.

### 2.8. Side-chain rebuilding

The side-chain building algorithm in *Buccaneer*, while fast, sometimes leads to incorrect conformations. To address this, a more flexible side-chain rebuilding step is performed once at the end of the pipeline if the resolution is better than 2.5 Å and *R*_work_ is better than 30%. The step rebuilds side chains that are either missing or predicted to be incorrect by the neural networks from the pruning step. Residues with a side-chain correctness less than 0.25 times the median and a main-chain correctness more than 0.25 times the median are selected. The rebuilding uses functions in *Coot* and starts by truncating the side chain back to C^β^ and then refining the residue along with its neighbours. This aims to improve the C^α^ and C^β^ positions without them being pulled out of place by refining an incorrect rotamer. The side chain is then re-added, the auto-fit-best-rotamer function is used to choose the rotamer and a final refinement of the residue and its neighbours is carried out. The model with rebuilt side chains is refined with five cycles of *REFMAC* and accepted if it has a better *R*_free_.

## 3. Test sets

### 3.1. Experimental phasing

A search was performed using the PDBe REST API for entries from the Joint Centre for Structural Genomics (JCSG; Elsliger *et al.*, 2010 ▸) where experimental X-ray data were available and the structure-determination method was listed as SAD or MAD. For each of the 1322 entries identified in this search, observations, a free-*R* flag and experimental phases were extracted from the deposited data. If the deposited data did not contain mean amplitudes then they were derived from anomalous amplitudes or intensities using the *CCP*4 program *CTRUNCATE* (French & Wilson, 1978 ▸). If the free-*R* flag had no zero values or more than 50% zero values then a new flag was generated using the *FREERFLAG* program (Brünger, 1997 ▸). The deposited structure was then refined using ten cycles of *REFMAC* after removing UNL residues and any atoms that did not agree with the monomer library. Entries were discarded if there was an error during processing, *R*_free_ was deemed to be too high (>0.06 × resolution/Å + 0.17), the data completeness was less than 90% or the *F*-map correlation between the experimental phases and the refined deposited structure was less than 0.2. *F*-map correlation is the correlation coefficient between the structure-factor amplitudes of the two maps weighted by the cosine of the phase difference. Table 1 ▸ shows the number of models discarded at each stage, leaving 1180 entries in the test set.

### 3.2. Molecular replacement

A set of 1351 placed molecular-replacement solutions was obtained from Bond *et al.* (2020 ▸). As described in the supporting information to that publication, PDB entries were randomly selected so that there was an even spread from 1 to 3.5 Å resolution and no two chains had a sequence identity of 50% or greater. Structures were only considered if the *MolProbity* (Chen *et al.*, 2010 ▸) clashscore, Ramachandran outliers and side-chain outliers and *EDS* (Kleywegt *et al.*, 2004 ▸) real-space *R*-value *Z*-score were in the top 40th percentile and the *DCC* (Yang *et al.*, 2016 ▸) *R*_free_ was in the top 50th percentile relative to structures at similar resolution. For each entry, the authors identified structural homologues using *GESAMT* (Krissinel & Uski, 2017 ▸) with sequence identity less than 70%, an r.m.s.d. of less than 3 Å and a *Q*-score of greater than 0.2. Models were prepared using *Sculptor* (Bunkóczi & Read, 2011 ▸) and molecular replacement was carried out using *Phaser* (McCoy *et al.*, 2007 ▸). They then chose a single model for each entry, ensuring that there was a spread in the *F*-map correlation (between the refined MR model and the refined deposited structure) between 0.2 and 0.9. Models with low *F*-map correlations may be a result of low structural similarity between the target and homologue, of the modelled entity being only a small component of the asymmetric unit or of incomplete or inaccurate molecular-replacement solutions. Each entry was processed in the same way as the experimental phasing test set by obtaining observed amplitudes and a free-*R* flag and refining the deposited structure. Table 2 ▸ shows the number of models discarded at each stage, leaving 1341 entries in the test set.

### 3.3. AlphaFold

*AlphaFold* (version 2) models for the human proteome (UP000005640) were downloaded in mmCIF format from AlphaFoldDB (Tunyasuvunakool *et al.*, 2021 ▸). Only the 20 294 models that were represented in a single file were considered. For each *AlphaFold* model, the PDB was searched for entries where the only polymer was an unmodified protein of at least 20 residues with the same UniProt accession. Entries also had to have experimental X-ray data available with a resolution of 4 Å or better.

For each PDB entry that was found in the search, a similarity was calculated by truncating the *AlphaFold* model to the observed residues, superposing the truncated model over the best chain using *Gemmi* and calculating the percentage of C^α^ atoms in the chain that are within 1 Å of a C^α^ atom in the *AlphaFold* model. The PDB entry with the lowest superposed similarity between 20% and 90% was chosen. This ensures that the test set only contains models that need some rebuilding and are not completely dissimilar.

The deposited structure and data were then downloaded, processed and assessed in the same way as the other test sets. Four search models were produced: one with the *AlphaFold* model truncated to the observed residues and three more where residues with pLDDT scores below 50, 70 and 90 were also removed. Molecular replacement was performed using *MOLREP* (Vagin & Teplyakov, 2010 ▸) and the solution with all copies placed and the best MR score was chosen. The MR score is the product of the correlation coefficient and the packing function, which is 1 if no molecules overlap and −1 if the molecules overlap completely. The result is only accepted into the test set if the *F*-map correlation is 0.2 or higher. Table 3 ▸ shows the number of models discarded at each stage, leaving 2031 entries in the test set.

## 4. Methods

*ModelCraft* and the *CCP*4*i* *Buccaneer* pipeline were run on each case in the three test sets. To prepare the data for the *CCP*4*i* *Buccaneer* pipeline, experimental phases were modified using five cycles of *Parrot* and models in the MR and *AlphaFold* test sets were refined with ten cycles of *REFMAC*. This was not performed for *ModelCraft* as density modification and model refinement are performed internally. Both pipelines were made to use the experimental phases in MLHL refinement with *REFMAC*, although *ModelCraft* stops using them once *R*_work_ decreases to 35%. An ablation study was also carried out on the molecular-replacement test set where individual steps in the *ModelCraft* pipeline were removed to see the effect on the overall performance. An additional test was carried out to reduce the number of cycles in *ModelCraft* from 25 to five, as was used in the original *CCP*4*i* *Buccaneer* pipeline.

Performance was measured using *R*_work_, *R*_free_, protein model completeness and the time it took the pipeline to finish. Completeness was calculated by moving the built model over the deposited model using *CSYMMATCH* and then taking the percentage of protein residues in the deposited model that had a matching residue in the built model. A residue was considered to match if N, C^α^ and C were all within 1 Å. *CSYMMATCH* uses the space-group symmetry operations to try and match each chain onto the reference model. It also accounts for possible differences in the cell origin, which is important as the molecular-replacement solutions are not guaranteed to have the same origin as the deposited model. Completeness is used as the primary metric for comparison between pipelines. Using *R* factors would be a less fair comparison because the *CCP*4*i* *Buccaneer* pipeline only builds protein and *ModelCraft* also builds nucleic acids and waters.

## 5. Results

**Figure 1:**
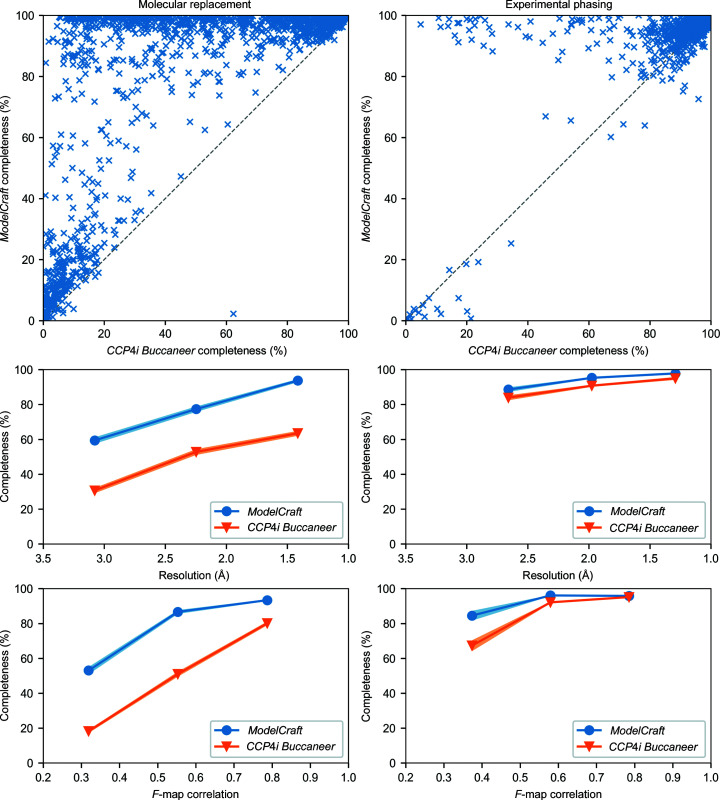
Comparison of *ModelCraft* and the *CCP*4*i* *Buccaneer* pipeline for molecular replacement (left) and experimental phasing (right). The top row shows protein completeness for each structure and the bottom rows show completeness as a function of resolution and *F*-map correlation. Structures were split into three resolution and *F*-map correlation bins. Points show the mean completeness at the centre of each bin and the shaded area shows one standard error either side.

Three cases from the molecular-replacement test set and one case from the *AlphaFold* test set were omitted from this analysis due to failures in either *CCP*4*i* *Buccaneer* or *ModelCraft*. Fig. 1 ▸ shows the completeness of the *ModelCraft* and *CCP*4*i* *Buccaneer* models for the molecular-replacement and experimental phasing test sets. *ModelCraft* produced a more complete model for 1290 (96%) of the 1338 molecular-replacement cases. It built 1000 cases (75%) to above 80% completeness, whereas the *CCP*4*i* pipeline only built 432 cases (32%) to above 80% completeness. There are also 189 cases (14%) where the completeness from both pipelines is below 20%. The experimental phasing test set was easier, with 1071 (91%) of the 1180 cases being built to above 80% completeness by both pipelines. Even so, *ModelCraft* builds a more complete model in 870 cases (74%). Both pipelines perform better when the data resolution and phase quality are higher. For the molecular-replacement test set, the mean *ModelCraft* completeness at 3 Å resolution is similar to the mean *CCP*4*i* *Buccaneer* completeness at 1.5 Å resolution. Both pipelines performed well on the experimental phasing test set so the difference is smaller for this set. The molecular-replacement test set was constructed to have a roughly even spread of resolutions between 1 and 3.5 Å and *F*-map correlations between 0.2 and 0.9. The experimental phasing test set has fewer cases with low resolutions and poor phases. However, it appears that *ModelCraft* performs better than the *CCP*4*i* *Buccaneer* pipeline when starting from poor experimental phases.

**Figure 2:**
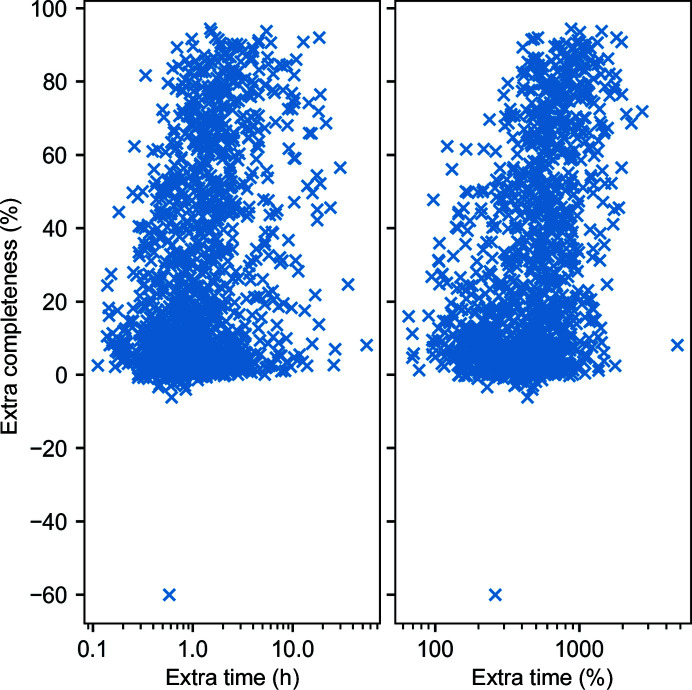
Extra completeness gained by using *ModelCraft* instead of the *CCP*4*i* *Buccaneer* pipeline against the extra time that it takes for the pipeline to finish for the molecular-replacement test set.

Fig. 2 ▸ shows the extra completeness gained from using *ModelCraft* against the extra time that it takes to run compared with the *CCP*4*i* *Buccaneer* pipeline. The time comparison is not exact because user CPU time was measured for the *CCP*4*i* *Buccaneer* pipeline but elapsed real time was measured for *ModelCraft* because it includes programs that do not report CPU time. Competition for resources between pipelines executed in parallel is likely to cause the elapsed real time to be longer. However, it is expected that *ModelCraft* will always be slower as the *CCP*4*i* pipeline runs for five cycles and *ModelCraft* runs for a minimum of five cycles with more steps included in each cycle. The mean amount of extra time is 2 h 1 min, from 21 min in the *CCP*4*i* pipeline to 2 h 23 min in *ModelCraft*. The median extra time is 1 h 6 min. The mean extra completeness is 28 percentage points, from 50% in the *CCP*4*i* pipeline to 78% in *ModelCraft*. The median extra completeness is 16 percentage points.

**Figure 3:**
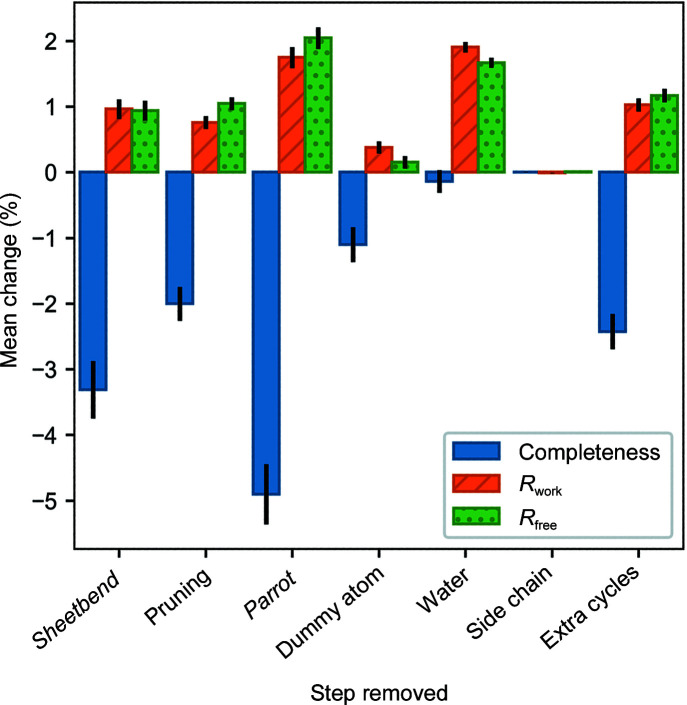
The mean change in completeness, *R*_work_ and *R*_free_ when individual steps are removed from the *ModelCraft* pipeline for the molecular-replacement test set. Error bars show one standard error above and below the mean.

Fig. 3 ▸ shows the mean change in completeness, *R*_work_ and *R*_free_ on removing individual *ModelCraft* steps for the molecular-replacement test set. Other than the final side-chain rebuilding step, removing any of the steps causes a degradation in average performance. The *Parrot* density-modification step has the largest effect when removed, making the average *R* factors and completeness much worse. Reducing the number of cycles from 25 to five has an effect of a similar magnitude to removing the initial *Sheetbend* refinement or the pruning steps. The mean changes come from distributions where the majority of test cases are unaffected but some have changed greatly, for example from a model that is mostly correct to a model of mostly incorrect fragments. Removing the side-chain rebuilding step makes the least difference, but this is expected as it is only performed once at the end of the pipeline in a subset of cases and only a small number of atoms are affected. This analysis does examine the interdependence between steps, so the effect of removing multiple steps at the same time is unknown.

**Figure 4:**
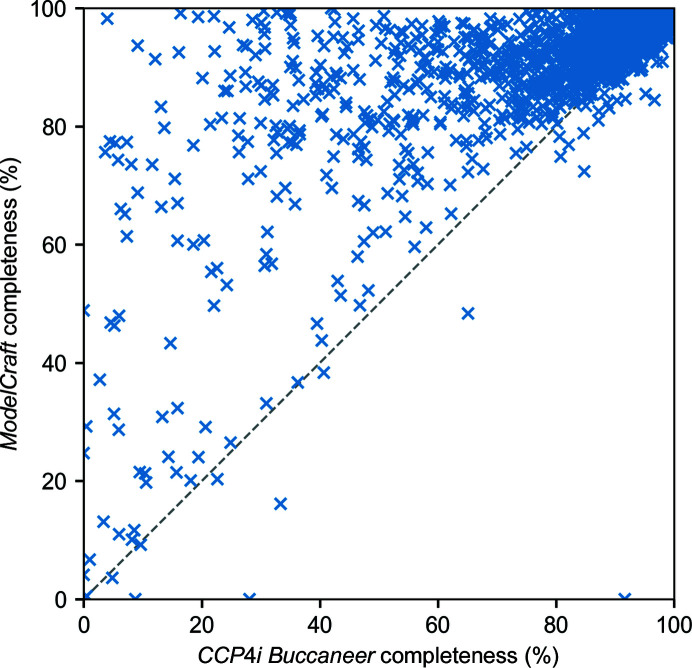
Completeness of the protein structure built by the *ModelCraft* and *CCP*4*i* *Buccaneer* pipelines for the *AlphaFold* test set.

Fig. 4 ▸ shows the completeness of the *ModelCraft* and *CCP*4*i* *Buccaneer* models in the *AlphaFold* test set. *ModelCraft* also shows good performance on this test set, building a more complete model than *CCP*4*i* *Buccaneer* in 1750 (86%) of 2030 cases. The structure with the greatest improvement is PDB entry 6wku (Boudko *et al.*, 2021 ▸), which is a single 695-residue chain that links together collagen IV α3, α4 and α5. The *AlphaFold* model for collagen IV α5 (UniProt P29400) was truncated to the 224 residues that make up the C-terminus of this chain. Residues with pLDDT values below 90 were removed and molecular replacement produced a solution with an *F*-map correlation of 0.45. *ModelCraft* managed to build the missing parts of α5 and the additional α3 and α4 regions to give a completeness of 98%, but *CCP*4*i* *Buccaneer* only achieved a completeness of 4% after losing the correct residues in the fragmented starting model. In the bottom right of Fig. 4 ▸, PDB entry 1dfn (Hill *et al.*, 1991 ▸) has a completeness of 92% with *CCP*4*i* *Buccaneer* and 0% with *ModelCraft*. It is a small structure with two copies of a 30-residue chain. Molecular replacement of the *AlphaFold* model (without a pLDDT cutoff) gave a solution with an *F*-map correlation of 0.95 that needs little rebuilding. Unfortunately, it is a rare case where the lack of restraints in shift-field refinement causes the input model to become scrambled before it is used by *ModelCraft*. The negative performance outlier in the molecular-replacement test set is a similar example where refinement of the molecular-replacement model with *REFMAC* alone leads to *R*_work_ and *R*_free_ values of 45% and 50%, respectively, but refinement with *Sheetbend* and then *REFMAC* worsens this to 52% and 55%, respectively. These failures either need to be addressed in *Sheetbend* or detected by *ModelCraft* so that an alternate refinement method can be used.

**Figure 5:**
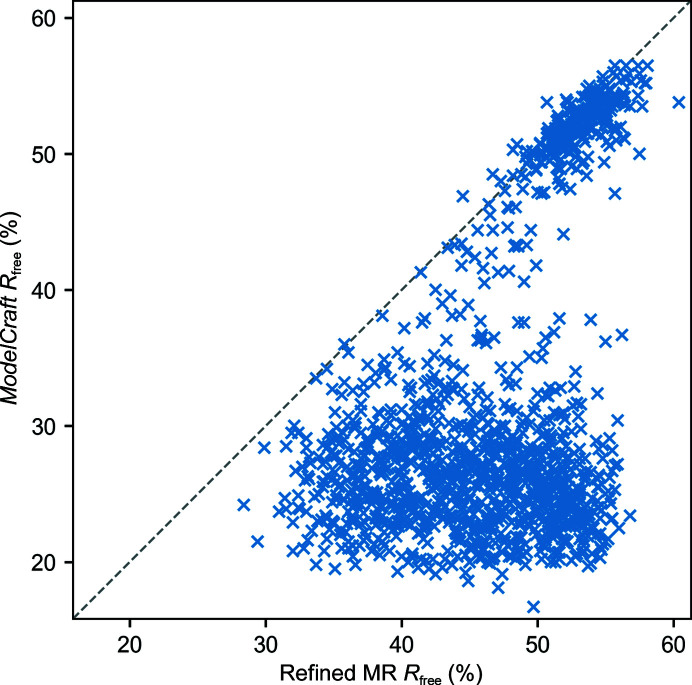
*R*_free_ after refining the starting model with *Sheetbend* and then *REFMAC* and *R*_free_ of the autobuilt structure from *ModelCraft* for the molecular-replacement test set.

The completeness metric used in this study is useful for comparing the two pipelines, but it cannot be calculated without reference to the completed model. To show the results that may be expected from *ModelCraft* with a widely available metric, Fig. 5 ▸ compares *R*_free_ after refining the starting model and at the end of *ModelCraft* for the molecular-replacement test set. Molecular-replacement models with *R*_free_ values below 50% are very likely to be improved by *ModelCraft*. However, there is a cluster of models with high *R*_free_ values that were not corrected. The likelihood that *ModelCraft* will improve a molecular-replacement model depends on many factors, such as data quality, resolution, structural similarity, the number of NCS copies, whether all chains are correctly placed and whether there are missing components.

## 6. Discussion

*ModelCraft* builds more complete models than the *Buccaneer* pipeline in *CCP*4*i*, especially when starting from poor or partial molecular-replacement solutions. *Sheetbend* uses large concerted shifts to refine the model or its placement, the initial pruning step removes incorrect parts of the model, and then *Parrot* and the addition of dummy atoms improve the phases to make it more likely that the corrected conformation will be built. It is common for *Buccaneer* to also build short protein fragments into the solvent region when the model is approaching completion (Alharbi *et al.*, 2019 ▸), but the chain-pruning step aims to address this. Lastly, building nucleic acids, waters and rebuilding side chains helps to finalize the structure and saves time during subsequent interactive building.

The downside of adding these steps is that it takes more time for the pipeline to run. For cases where speed is important, *ModelCraft* includes an option to run a basic pipeline that only includes *Buccaneer*, *Nautilus* and *REFMAC* in each cycle. Increasing the default number of cycles from five to 25 and choosing the model from the cycle with the lowest *R*_free_ were also factors in the improved average completeness. There is a trade-off between completeness and computational cost when choosing the number of cycles to run. Some structures will be completed in the first couple of cycles, some need more than 25 cycles and some the pipeline may never be able to complete. The automatic stopping function was introduced to make this choice easier by stopping the pipeline if *R*_free_ has not improved for four cycles. However, this could be improved by replacing *R*_free_ with completeness metrics. If the whole structure has been built with good geometry and fit to density then the pipeline could be stopped immediately. Using these metrics would also make it easier to develop a more intelligent pipeline that tries the quickest methods first and only uses slower steps if they are needed.

Although this study has focused on protein model building, *ModelCraft* also includes *Nautilus* for building nucleic acids. Future work is planned to produce a test set that includes nucleic acids and to improve nucleic acid building in *ModelCraft*. The *Parrot* density-modification and dummy-atom addition steps are expected to be beneficial to both protein and nucleic acid structures. However, the current pruning steps only remove protein residues, so they will not improve the correction of mistakes in the nucleic acid structure. This is especially important for structures with a mixture of protein and nucleic acids as *Nautilus* may build nucleotides into the protein density and prevent *Buccaneer* from building there in subsequent cycles.

Molecular-replacement models and experimental phases are not often made available, so the test sets used here are not fully representative of user data. The experimental phasing test set uses data from the automated JCSG pipeline (Elsliger *et al.*, 2010 ▸), which may differ from more recent data-collection, processing and phasing methods. The molecular-replacement test set contains partial solutions where a user would probably repeat molecular replacement to place more components. The *AlphaFold* test set does not split the predicted models into high-confidence regions, which can lead to either poor solutions or molecular replacement failing when there are errors in the relative domain positions. However, the goal was not to create the best initial models but to assess the ability to build a complete structure from a range of starting points.

Only structures solved by X-ray crystallography were used in this study. *ModelCraft* can also build into cryo-EM maps using the changes to the *Buccaneer* and *REFMAC* options described by Hoh *et al.* (2020 ▸). The automatic stopping function then measures improvement using average Fourier shell correlation (FSC) instead of *R*_free_. However, the cryo-EM pipeline only includes *Buccaneer*, *Nautilus* and *REFMAC*, so its performance is expected to be similar to the existing *Buccaneer* pipeline in the *CCP-EM* suite (Burnley *et al.*, 2017 ▸). Although the phase-improvement steps used for X-ray data are not applicable, the pruning steps should be modified to work for both methods.

## 7. Availability

*ModelCraft* is available in *CCP*4 8.0 both from the command line and from graphical interfaces in *CCP*4*i*2 and *CCP*4 Cloud. All scripts used for preparing the test sets, running the pipelines and analysing the results can be found at https://doi.org/10.5281/zenodo.6856249. 

## Figures and Tables

**Table 1:** Entries discarded from the experimental phasing test set

Count	Reason
36	No experimental phases deposited
13	Different cell or space group in the structure and data
1	Error during *REFMAC*
45	Data completeness less than 90%
37	*R*_free_ deemed to be too high (>0.06 × resolution/Å + 0.17)
10	*F*-map correlation less than 0.2

**Table 2:** Entries discarded from the molecular-replacement test set

Count	Reason
1	Entry has been obsoleted in the PDB
1	Different cell or space group in the structure and data
3	Data completeness less than 90%
4	*R*_free_ deemed to be too high (>0.06 × resolution/Å + 0.17)
1	Recalculated *F*-map correlation less than 0.2

**Table 3:** Entries discarded from the *AlphaFold* test set

Count	Reason
16427	No PDB entries
1335	No PDB entries with superposed similarity between 20% and 90%
9	Error processing structure-factor data
3	Different cell or space group in the structure and data
185	Data completeness less than 90%
151	*R*_free_ deemed to be too high (>0.06 × resolution/Å + 0.17)
82	Molecular replacement could not place all copies
71	*F*-map correlation less than 0.2
